# Starch-Based Film Coatings as a Strategy to Preserve Seed Viability during Storage

**DOI:** 10.1021/acsomega.6c01438

**Published:** 2026-06-20

**Authors:** Giovana A. Parolin, Matheus C. R. Miranda, Tereza S. Martins, Marystela Ferreira, Laura O. Péres

**Affiliations:** † Laboratory of Hybrid Materials–Chemistry Department, Federal University of São Paulo, Diadema, SP 09913-030, Brazil; ‡ Science and Technology Center for Sustainability, Federal University of São Carlos, Sorocaba, SP 18052-780, Brazil

## Abstract

Seed viability progressively declines during storage, and many existing coating strategies either impair germination or rely on synthetic, nonbiodegradable materials. In this work, we present a sustainable, starch-based film coating as a proof-of-concept strategy to preserve seed viability under monitored ambient storage conditions. Corn starch films plasticized with glycerol and urea were prepared and screened, leading to the selection of two formulations (A3 and A8) with distinct physicochemical properties. Tomato seeds were used as a sensitive model system and evaluated after 60, 150, and 240 days of storage. Both coatings effectively mitigated the pronounced decline in germination and vigor observed for uncoated seeds. The more compact and thermally stable A3 formulation provided superior long-term protection, while the more hydrophilic A8 film exhibited faster dissolution, promoting early seedling development. Physicochemical characterization indicates that the coatings act as semipermeable barriers during storage, reducing water and oxygen diffusion, followed by hydration-driven swelling and partial dissolution during germination. Although demonstrated at laboratory scale, these results highlight the feasibility of tuning starch-based film properties to balance storage protection and functional dissolution. This work establishes a biodegradable, low-cost, and environmentally friendly platform for the development of next-generation seed coating systems with potential applicability across diverse crop species.

## Graphical abstract

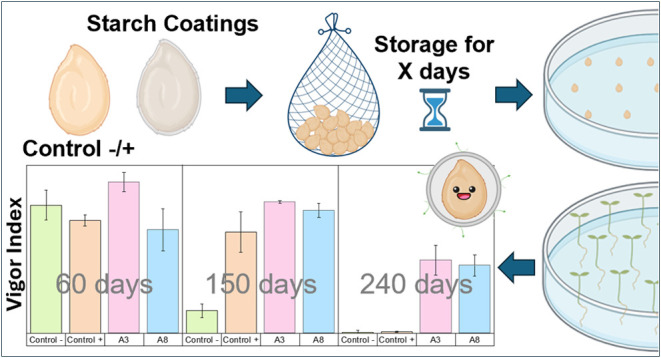

## 1 Introduction

Seed coating is a promising technique for enhancing seed performance by improving germination rates, shelf life, quality, yield, and overall efficacy through the modulation of cell membrane permeability, cellular respiration, metabolic activity, and other physiological processes. It consists of coating the seeds with specific materials that modify their physical properties, thereby improving handling, quality, and performance. One of the main advantages of this method is the enhancement of seed resistance to external factors, such as extreme temperatures, environmental conditions, exposure to chemical agents or heavy metals, and microorganisms. Some drawbacks may include delayed germination, incompatibility between the coating material and the seed, and increased costs.^1,2^

In this context, biopolymers emerge as a promising alternative to overcome some of these challenges, representing a sustainable solution due to their biodegradability, bioavailability, and biocompatibility. Among them, starch, a polysaccharide, stands out as an attractive and cost-effective material due to its excellent film-forming ability, abundance, nontoxicity, barrier properties, accessibility, and others. However, the incorporation of plasticizing agents, such as glycerol, polyethylene glycol, natural plant extracts, citric acid, formamide, urea, or their combinations, is often required to enhance the thermal and mechanical properties of starch-based films, particularly their flexibility and resilience.^3–9^ These films are commonly employed in food packaging, and several studies have also reported their use in the protection of fruits, vegetables, and other perishable products.^10–13^

Tomato seeds, for example, have high commercial value and, if not stored under controlled conditions, particularly with respect to moisture and temperature, may lose their physiological integrity, resulting in reduced viability and vigor.^14,15^ Additionally, tomato seeds are an excellent model system due to their pronounced sensitivity to environmental changes, enabling a reliable assessment of storage-related effects. According to Corbineau, the half-viability period (*P*_50_) of tomato seeds can reach approximately 20 years; however, this estimate assumes storage under strictly controlled environments (temperate climate, low temperatures, and high relative humidity),^14^ which clearly does not reflect the reality faced by many agricultural workers.

In this context, the development of biodegradable, nontoxic, natural, widely available, and, above all, low-cost materials for seed protection represent an accessible, simple, promising, and emerging alternative to extend seed storage time. Although several studies have reported the use of coatings for tomato fruits in postharvest preservation,^11,16,17^ there is still limited information regarding their application to tomato seeds. Therefore, this study aimed to develop biodegradable corn starch-based films for tomato seed coating, investigating their physicochemical properties and evaluating their effectiveness in preserving seed viability and vigor during storage, with long-term efficacy.

## 2 Materials and Methods

### 2.1 Materials

Commercially available corn starch and tomato seeds (*Solanum lycopersicum*), purchased from a local market, were used without further purification. Glycerol (Sigma-Aldrich, ≥99.5%), commercial sodium hypochlorite (Candida, 2.0–2.5% w/w), urea (Synth, ≥99.0%), and silica gel (Synth, 2–4 mm) were also used as received.

### 2.2 Preparation and Characterization of Corn Starch Films

To prepare the starch films, the plasticizing agents urea and glycerol were used to improve the physical, chemical, and mechanical properties of these films. Accordingly, samples with the following compositions were prepared and visually analyzed for their feasibility as seed coating materials (Table 1). The film preparation was based on the methodology reported in the literature,^18^ which was adapted and further optimized through systematic experimental adjustments to achieve the most suitable formulation for this intended application. With that, a volume of 33.4 mL of water was heated to 70–80 °C, and the corresponding mass of starch was added. After 10 min of stirring, glycerol was added, and the mixture was stirred for an additional 20 min, followed by the addition of urea, with continued stirring for another 10 min. The resulting solution was then allowed to cool to room temperature, cast for drying, and subsequently placed in an oven at 40 °C for 48 h (Figure 1).

**Figure 1:**
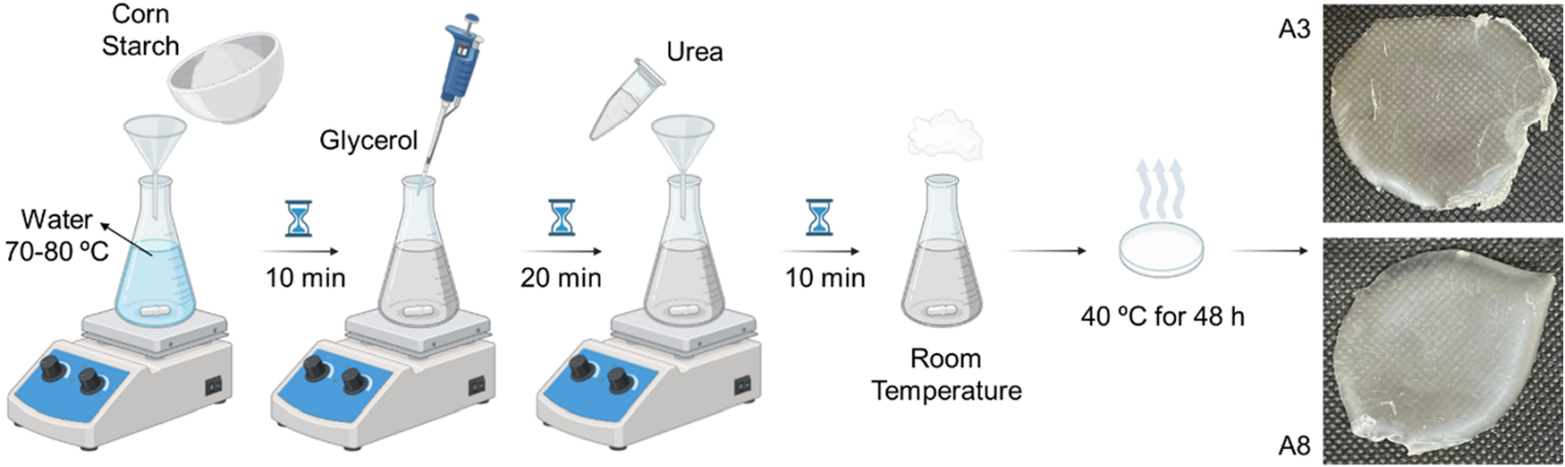
Schematic preparation of corn starch films.

**Table 1:** Composition of the Starch-Based Films Prepared, Including the Amount of Each Component in the System in % (w/w)

Sample	Corn Starch (%)	Glycerol (%)	Urea (%)
A1	84	16	-
A2	78	22	-
A3	60	17	23
A4	66	19	15
A5	55	16	29
A6	73	0	27
A7	68	6	26
A8	54	26	20
A9	100	0	0

Based on a preliminary evaluation, two formulations, A3 and A8, were selected as the most suitable and subsequently used for coating the seeds. Furthermore, these two films were characterized by contact angle measurement, thermogravimetric analysis (TGA), Fourier-Transform Infrared spectroscopy (FTIR), and scanning electron microscopy (SEM).

Additionally, the environmental friendliness of the film preparation strategy was evaluated using the AGREE (Analytical GREEnness) metric, which integrates the principles of Green Analytical Chemistry into a unified sustainability score. Although originally developed for analytical methodologies, the AGREE tool was applied as a comparative indicator to assess the greenness of the corn starch-based coating preparation at the laboratory scale. Each of the 12 AGREE principles was qualitatively scored based on reagent toxicity, energy consumption, number of steps, waste generation, biodegradability, and operational safety, resulting in an overall greenness score.^19^

#### 2.2.1 Water Vapor Permeability Test

Water vapor permeability test was evaluated using sealed tubes containing a small orifice in the cap (contact area = 33.2 cm^2^). Each tube was filled with a known amount of silica gel and covered with corn starch-based films under investigation. Two control conditions were also included, with silica gel inside: one tube left open to the environment (environment control), and another sealed with aluminum foil (sealed control). All tubes were placed inside a desiccator containing water to create a saturated water vapor environment (Relative Humidity (RH) ranging from 81 to 88%). The assemblies were weighed at regular intervals over 120 h to monitor moisture uptake as an indicator of permeability (Figure 2). All experiments, adapted from the literature, were performed in triplicate to ensure reproducibility.^13,20^

**Figure 2:**
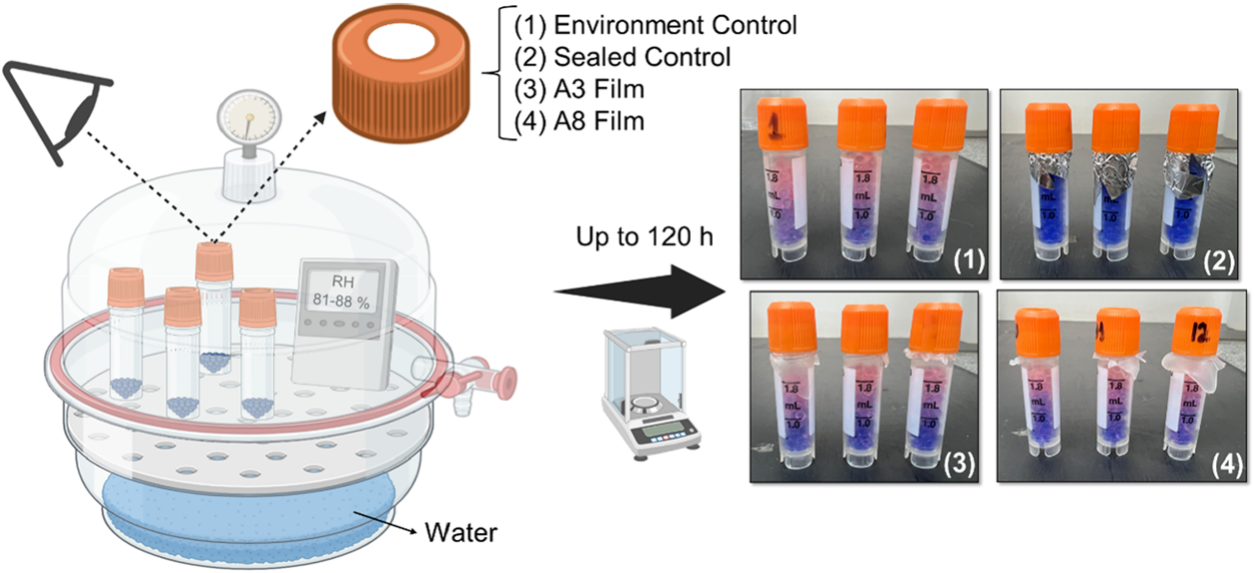
Schematic representation of the water vapor permeability test.

#### 2.2.2 Water Solubility Test

For the solubility test, precut film samples (1 cm^2^) were first dried in an oven at 40 °C to ensure accurate initial weight. Each sample was then placed in a Falcon tube containing 5 mL of water. At predetermined time intervals (ranging from 0 to 170 h), the samples (in triplicate) were removed, completely dried, and reweighed to determine the residual mass of the starch film.^13,20^

### 2.3 Preparation of the Seeds–Film Coating Procedure

The coating procedure followed the steps mentioned in Figure 3. Cherry tomato (*S. lycopersicum*) seeds were purchased and divided into four groups: a negative control group (which did not undergo any of the procedures described below), a positive control group (subjected only to water immersion), and two groups coated with starch-based films in two different formulations (A3 and A8). Before treatment, the last three groups were sanitized using sodium hypochlorite, followed by thorough rinsing with water to ensure complete cleaning of the seeds. The seeds were then immersed in either water, starch solution A3, or starch solution A8 for 24 h. After immersion, the seeds were individually placed on plastic Petri dishes and incubated in an oven at 30 °C for approximately 48 h, until complete drying was achieved.^21^ All samples were stored in small nonwoven fabric pouches for defined periods under ambient conditions, with average temperatures ranging from 17 to 26 °C and relative humidity between 70 and 88%.

**Figure 3:**
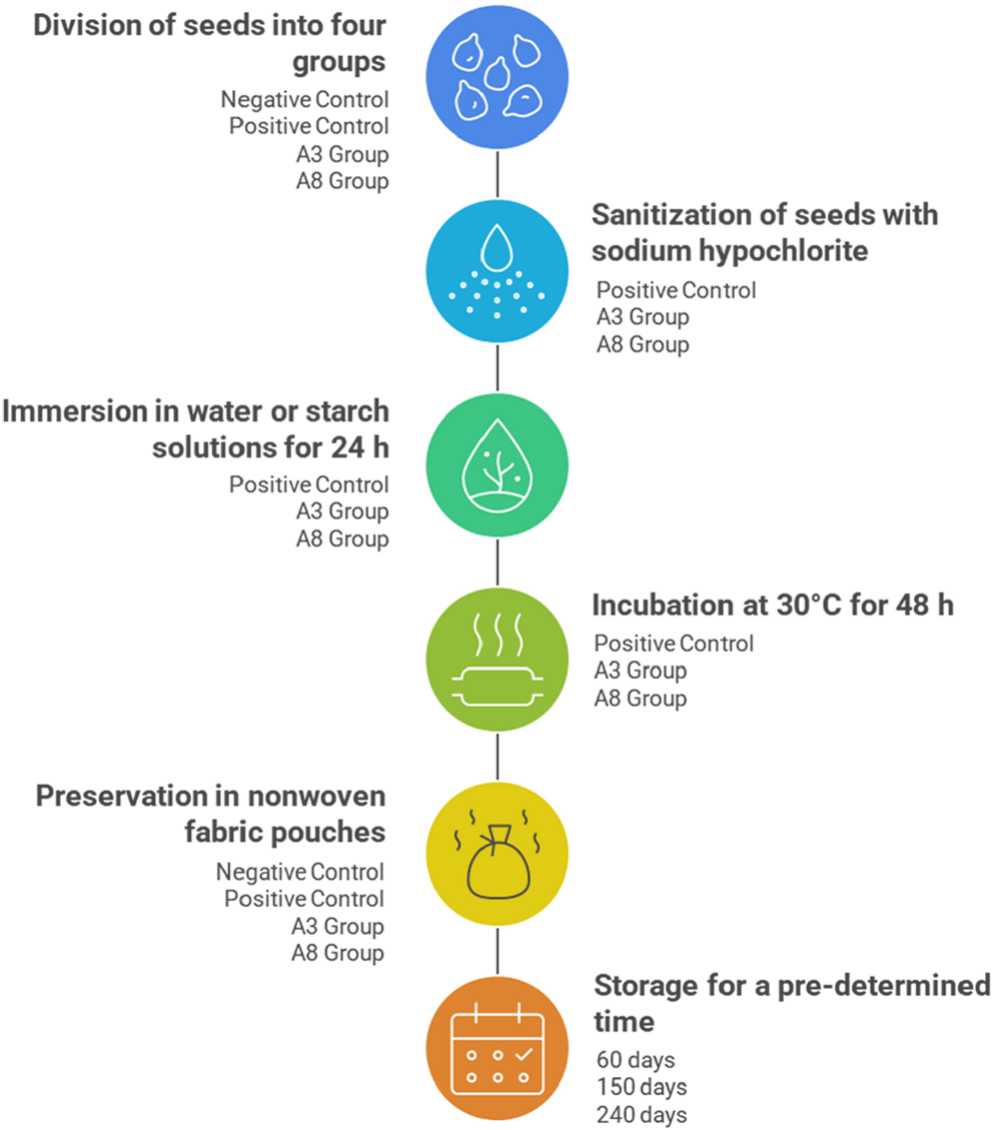
Overview of experimental steps.

### 2.4 Germination Assay

Germination assays were conducted at three different time points: the first, 60 days after seed coating; the second, 150 days after coating; and the third, 240 days postcoating to evaluate the influence of storage time on the germination efficacy. Each Petri dish was prepared with 15 seeds from the corresponding treatment group (positive and negative control, A3 and A8), and all tests were performed in triplicate. Petri dishes of 15 cm in diameter were previously cleaned with water and detergent, assembled using glass fiber filter and 15 mL of deionized water. The seeds were placed evenly over the moistened filter, and the dishes were tightly sealed with Parafilm. All plates were incubated in an oven at 28 °C for 7 days.^22^ After the incubation period, the plates were opened, and the seedlings were photographed and analyzed using ImageJ software, assessing shoot and root length, as well as leaf emergence. Germination and vigor indices were calculated according to (eqs 1 and 2) below

1 $$Ger\min ation\;Index\;\left(\%\right)=\frac{Number\;of\;Ger\min ated\;Seeds}{Total\;number\;of\;Seeds}\times 100$$

2 VigorIndex=GerminationIndex×SeedlingLength

where the seedling length refers to the sum of the lengths of the shoot and root.

### 2.5 Characterization and Instrumentation

Contact angle measurements were performed using a Model OCA 15 goniometer (Dataphysics) via the sessile drop method. A 4 μL droplet of deionized water was carefully deposited onto the film surface using a microsyringe. The contact angle was recorded at room temperature (∼25 °C) immediately after droplet deposition. For each sample, measurements were taken at three different positions, and the mean value was reported. Thermogravimetric analysis (TGA) and Differential Scanning Calorimetry (DSC) were performed using a Discovery SDT-650 equipment (TA Instruments). Approximately 14 mg of each film sample was heated from 30 to 1000 °C at a rate of 10 °C/min under a nitrogen atmosphere with a flow rate of 20 mL/min. Fourier-transform infrared (FTIR) spectra were performed using a Cary 630 FTIR spectrometer (Agilent) coupled to an ATR module (Attenuated Total Reflectance). Spectra were acquired in the range of 4000–400 cm^–1^ with 256 scans at a resolution of 4 cm^–1^. For scanning electron microscopy (SEM) images, the JSM-6610LV (JEOL) equipment was used, and the samples were sputter-coated with a thin layer of gold to improve conductivity. Observations were performed at an accelerating voltage of 10 kV under high-vacuum mode.

## 3 Results and Discussion

### 3.1 Preparation and Characterization of Corn Starch Films

Starch-based films were prepared with different compositions (Table 1) to evaluate their suitability for seed coating applications. Accordingly, the films were evaluated in terms of their visual appearance, mechanical integrity, and physicochemical properties. Figure S1 (Supporting Information) presents photographic images of the films, illustrating their overall appearance, which shows images of all tested formulations. Films A1, A2, and A6 exhibited considerable brittleness, which hindered their removal from the Petri dish after drying. Films A7 and A9 displayed high rigidity, with A7 also presenting pronounced opacity. Film A4 displayed marked mechanical fragility, while film A5 rapidly developed a porous structure. Based on these observations, formulations A3 and A8 were selected for further testing as promising candidates for detailed characterization and application studies.

Accordingly, FTIR was performed to verify whether the chemical structure of the material corresponded to the expected composition (Figure 4 (A)). A broad absorption band was observed at 3325–3335 cm^–1^, assigned to O–H stretching from hydroxyl groups present in starch and glycerol, as well as N–H stretching from urea, all of which are components of the film formulation. A distinct band at 2924 cm^–1^ is attributed to aliphatic C–H stretching. The absorption at 1625 cm^–1^ is assigned to C=O stretching from urea, along with possible contributions from residual water or O–H bending. A band at 1459 cm^–1^ corresponds to N–H bending vibrations. In the region from 997 to 1153 cm^–1^, bands were assigned to C–O and C–C stretching from the glucose rings in starch and C–O–C vibrations from both starch and glycerol. Finally, a signal at 924 cm^–1^ was attributed to ring deformation or torsion, characteristic of starch structures. The spectral bands observed are in good agreement with literature data,^4^ corroborating the presence of intermolecular interactions between the plasticizers and the starch matrix.

**Figure 4:**
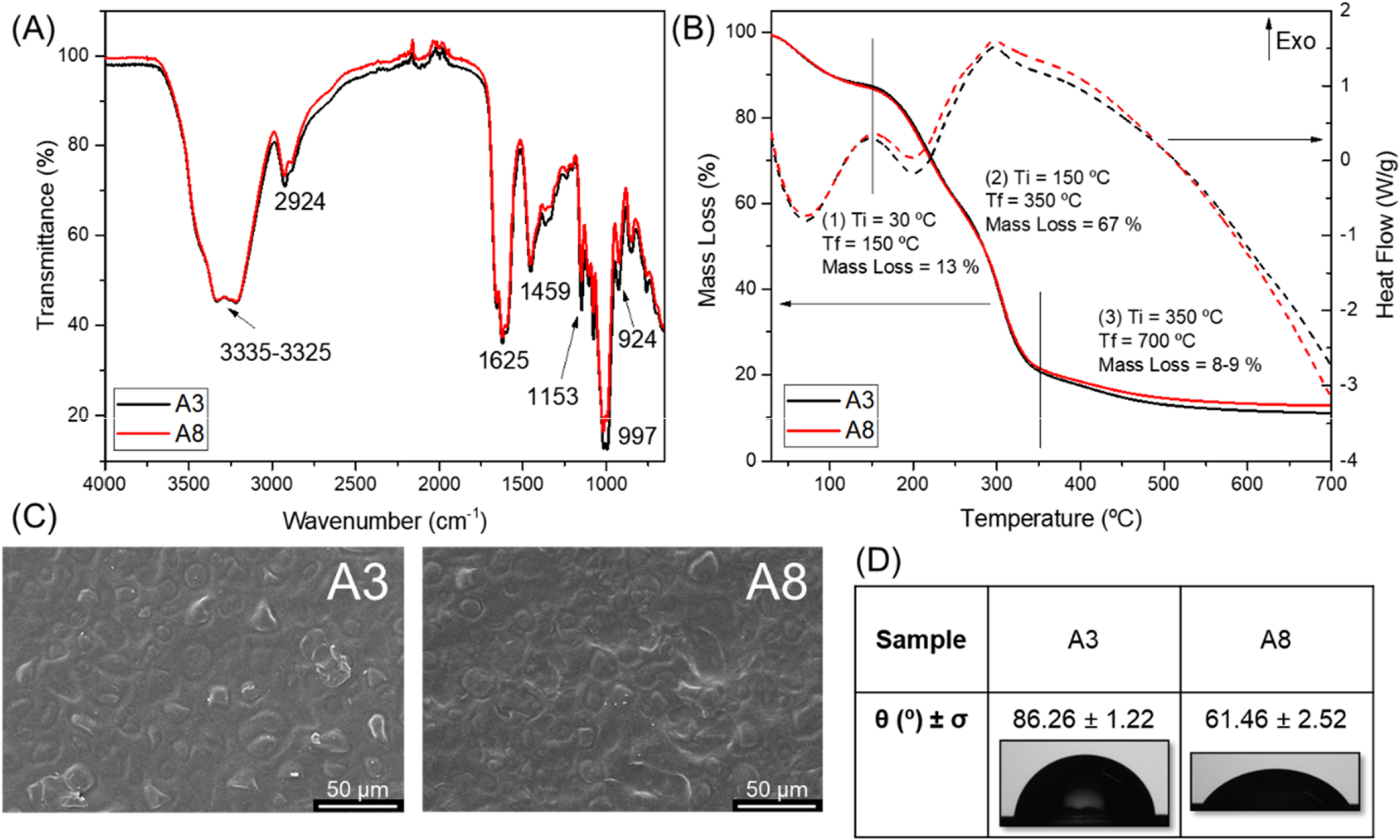
(A) FTIR spectra of A3 (black lines) and A8 (red lines) films; (B) TGA (solid lines) and DSC (dashed lines) analysis for A3 and A8 films; (C) SEM images (amplification ×500) and (D) contact angle values for formulations A3 and A8.

Thermogravimetric analysis of formulations A3 and A8 revealed similar thermal degradation profiles (Figure 4 (B)), characterized by three main stages: an initial mass loss (∼13%) up to 150 °C related to water evaporation, a significant degradation event between 150–350 °C (mass loss ∼ 67%) associated with the decomposition of urea, glycerol, and partial starch breakdown, and a final mass loss event above 350 °C corresponding to full starch degradation (∼8–9% mass loss).^4^ The DSC curves supported these findings, showing an endothermic event up to 150 °C due to water evaporation, corroborating the TGA results, followed by a prominent thermal transition between 150 and 300 °C, indicative of interactions among starch and plasticizers.^23^ While TGA curves exhibited nearly identical mass loss behavior, subtle differences were observed in the DSC profiles. These variations can be attributed to the higher plasticizer content and lower starch fraction in film A8. Specifically, the increased amounts of glycerol and urea weaken the intermolecular interactions within the starch matrix, reducing its thermal stability.^24^ As a result, in practical terms, the plasticizers evaporate at lower temperatures, which enhances chain mobility and is reflected as a slightly more pronounced thermal event in the DSC curve of film A8 compared to film A3.

The SEM images, presented in Figure 4(C), reveal that the A3 film presents a slightly more homogeneous surface, characterized by well-defined and rounded domains, which may indicate a greater compatibility among the components and a more uniform polymeric structure. In contrast, the A8 film exhibits a rougher and more irregular morphology, likely due to a higher plasticizer content leading to structural disorganization during film formation.^7^ Overall, the more stable and homogeneous structure of the A3 film suggests its greater potential for applications such as seed coating. Finally, the contact angle (θ) measurements (Figure 4(D)) were performed in triplicate, and the films A3 and A8 exhibited values of 86.26 ± 1.22° and 61.46 ± 2.52°, respectively, indicating an enhancement in the hydrophilicity, consistent with the increase in glycerol amount (from 17 to 26%).

Concerning sustainable terms, the AGREE-based assessment resulted in a high greenness score (∼0.78) (Figure S2—Supporting Information), reflecting the use of renewable and biodegradable materials, low-toxicity reagents, minimal waste generation, and an energy-efficient preparation process. This result reinforces the sustainability-oriented design of the proposed coating strategy, supporting its suitability as a proof-of-concept platform for the development of environmentally friendly seed coating systems.

### 3.1.1 Water Vapor Permeability and Solubility Tests

To assess the water vapor permeability (WVP) test, sealed tubes containing silica gel were used to monitor weight gain resulting from water absorption. The mass of the tubes was measured at different time points, up to 120 h of exposure. Relative WVP was also calculated, and the results are shown in Figure 5(A).

**Figure 5:**
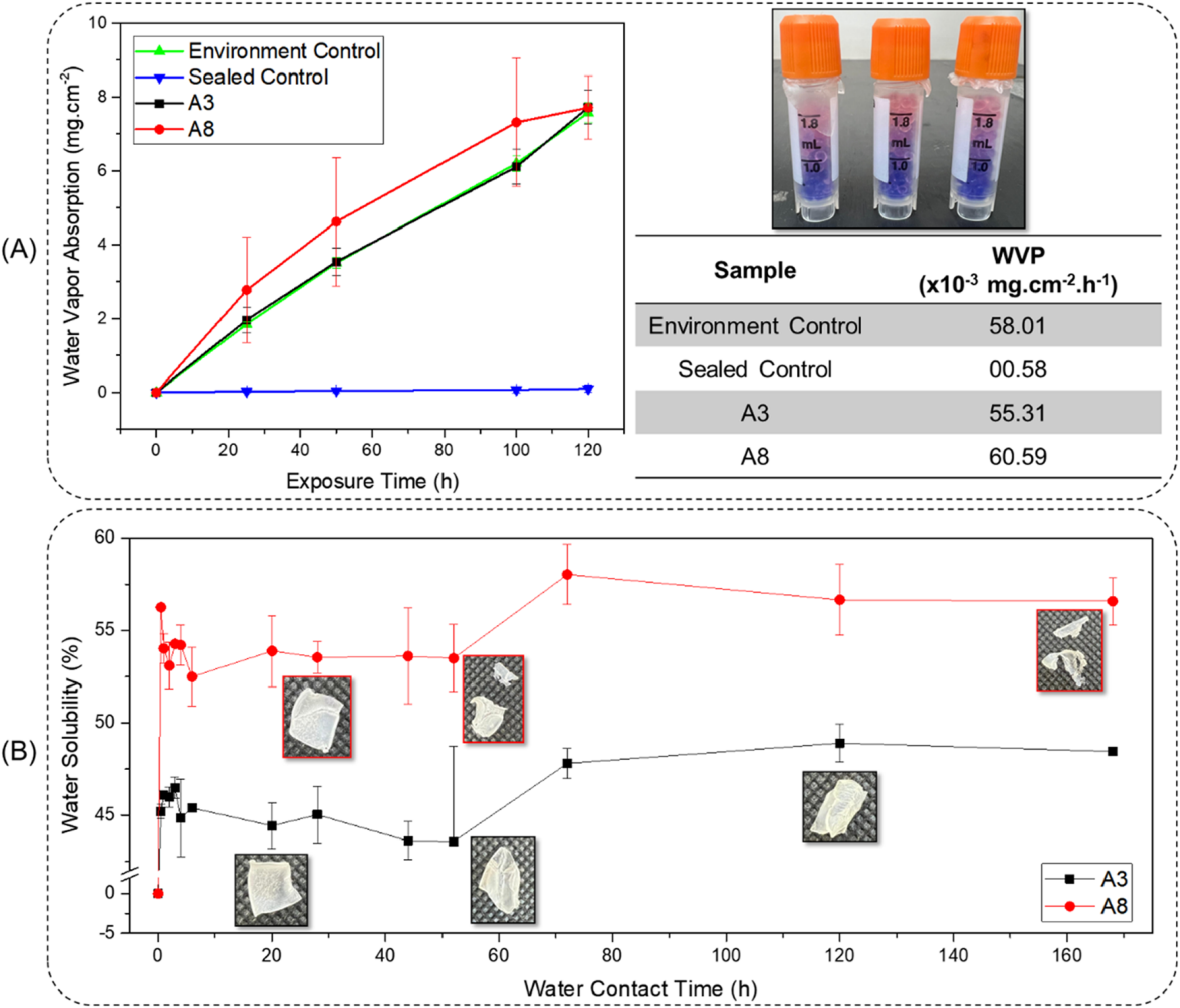
(A) Results from the Water Vapor Permeability (WVP) test over time and relative WVP values for each group, and (B) water Solubility test with both corn-starch formulations.

As expected, the control tube sealed with aluminum foil maintained a constant mass, indicating negligible permeability. The control tube open to the environment exhibited a mass variation profile similar to that observed for formulation A3. Notably, the highest water vapor absorption (and consequently the highest relative WVP) was observed for the A8 film. This behavior can be attributed to the higher glycerol content in A8, which likely increases WVP by enhancing matrix hydrophilicity and facilitating water diffusion due to its hygroscopic nature, as also supported by the lower contact angle values observed for this formulation. In contrast, formulation A3, with a higher starch content and reduced levels of plasticizers, showed an approximate 5.3% reduction in relative WVP. This is consistent with previous findings indicating a more compact and homogeneous matrix, reduced hydrophilicity, and enhanced thermal stability compared to A8. Consistent with these observations, previous studies have reported that higher concentrations of plasticizers, particularly glycerol, tend to weaken the attractive intermolecular forces within the polymer matrix.^20,25^ This reduction in cohesion may lead to a less dense and more disordered structure, thereby increasing water vapor permeability as a result of enhanced molecular mobility and polymer chain reorganization.

Concerning the water solubility test, samples of formulation A3 and A8 (1 cm × 1 cm) were cut, weighed, and immersed in distilled water inside Falcon tubes for up to 7 days (time of germination assay), in triplicate. At different exposure times, the samples were removed, dried, and reweighed. The results are presented in Figure 5(B).

Initially, the A8 film exhibits higher water solubility, reaching an average final value of 56.6%, compared to 48.4% for the A3 film. This behavior was anticipated, considering the greater hydrophilicity of the A8 formulation, which contains a higher concentration of glycerol. In contrast, the A3 film likely benefits from stronger intermolecular interactions and reduced water affinity in comparison to the other sample. The solubility profiles also reveal a plateau phase up to approximately 50 h, followed by a marked increase in solubilization until around 70 h, after which the values stabilized and remained approximately constant for the rest of the 7-day assay. The initial stage may be associated with the swelling of the polymer matrix, which precedes the progressive dissolution observed in the second phase. This final plateau suggests that the maximum solubility was achieved within the time frame of the experiment. Notably, as evidenced by the images, the residues of both samples exhibit distinct characteristics: while the A3 film remains nearly intact (indicating higher mechanical resistance), the A8 film shows visible signs of fragility after approximately 50 h, suggesting lower mechanical integrity upon hydration.^5^

Therefore, the A3 sample is expected to perform well in germination assays, acting as an effective physical barrier that protects seeds from microorganisms and moisture due to its favorable morphological, thermal, and mechanical properties. Although less mechanically robust than A3, the faster dissolution of A8 could be advantageous for applications requiring earlier seed hydration and germination, while still offering some initial protection compared to untreated seeds.

### 3.2 Tomato Seeds Coating with Corn Starch Films

Based on these results, tomato seeds were coated with both A3 and A8 formulations and subsequently characterized by SEM and TGA. TGA curves (Figure S3 and Table S1—Supporting Information) indicate that all samples exhibit similar thermal behavior, showing comparable mass loss steps and proportions. However, some points can be highlighted: (1) the treatments (whether soaked in water or coated with the formulations) do not significantly affect the moisture content, as the initial mass loss attributed to water evaporation is nearly identical for all samples; (2) the onset of significant thermal degradation occurs earlier for seeds coated with the A8 formulation than for those coated with A3. This aligns with the previously observed thermal profiles of the films, where A3 demonstrated superior thermal stability; (3) finally, the amount of residual mass supports the prior discussion: A3 leaves a higher amount of carbonaceous residue than A8, confirming that the formulation A3 presents a more thermally stable and compact structure.

Throughout the SEM images of both coated and uncoated seeds (Figure 6), it is possible to estimate the coating thickness: the A3 and A8 formulations exhibit average coverage of approximately 26.0 ± 3.1 μm and 43.6 ± 13.0 μm, respectively. Additionally, the A8 formulation exhibits a greater coating thickness compared to A3, which may directly influence its performance during the germination assay. It is perceived that positive control, immersed in water, (as well as the other coated seeds) demonstrates higher swelling in comparison to the negative (untreated) control. Moreover, the images reveal that the seed surfaces are effectively protected by the starch-based coating (mainly for the A3-coated seeds), forming a water-soluble physical barrier. This barrier is expected to prolong the shelf life of the seeds by offering temporary protection against moisture and microbial contamination, as will be further discussed.

**Figure 6:**
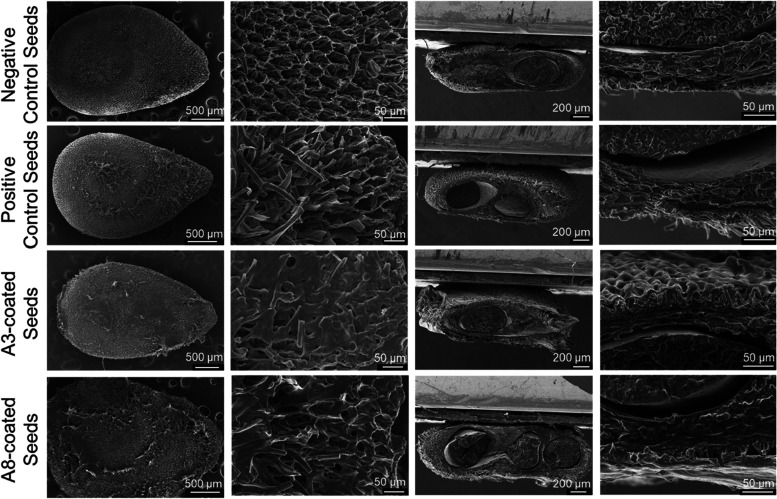
SEM images of the surface view and the cross-sectional area of the four distinctive groups: positive and negative control, and coated seeds with A3 and A8 films at different amplifications.

### 3.3 Germination Assay

The germination assay was conducted in triplicate to ensure reliability, with four groups: a negative control (untreated seeds), a positive control (seeds treated only with water), and seeds coated with formulations A3 and A8. After a 7-day incubation period at 28 °C, the seedlings were evaluated for shoot and root lengths, vigor and germination indices, and other important germination parameters, as moisture, dry and fresh weight, and leaf emergence. The assay was performed at three different periods: 60, 150, and 240 days after seed coating, to assess the long-term influence of the coating on germination efficiency (Figure S4—Supporting Information). The results are presented in Table 2 and Figure 7.

**Figure 7:**
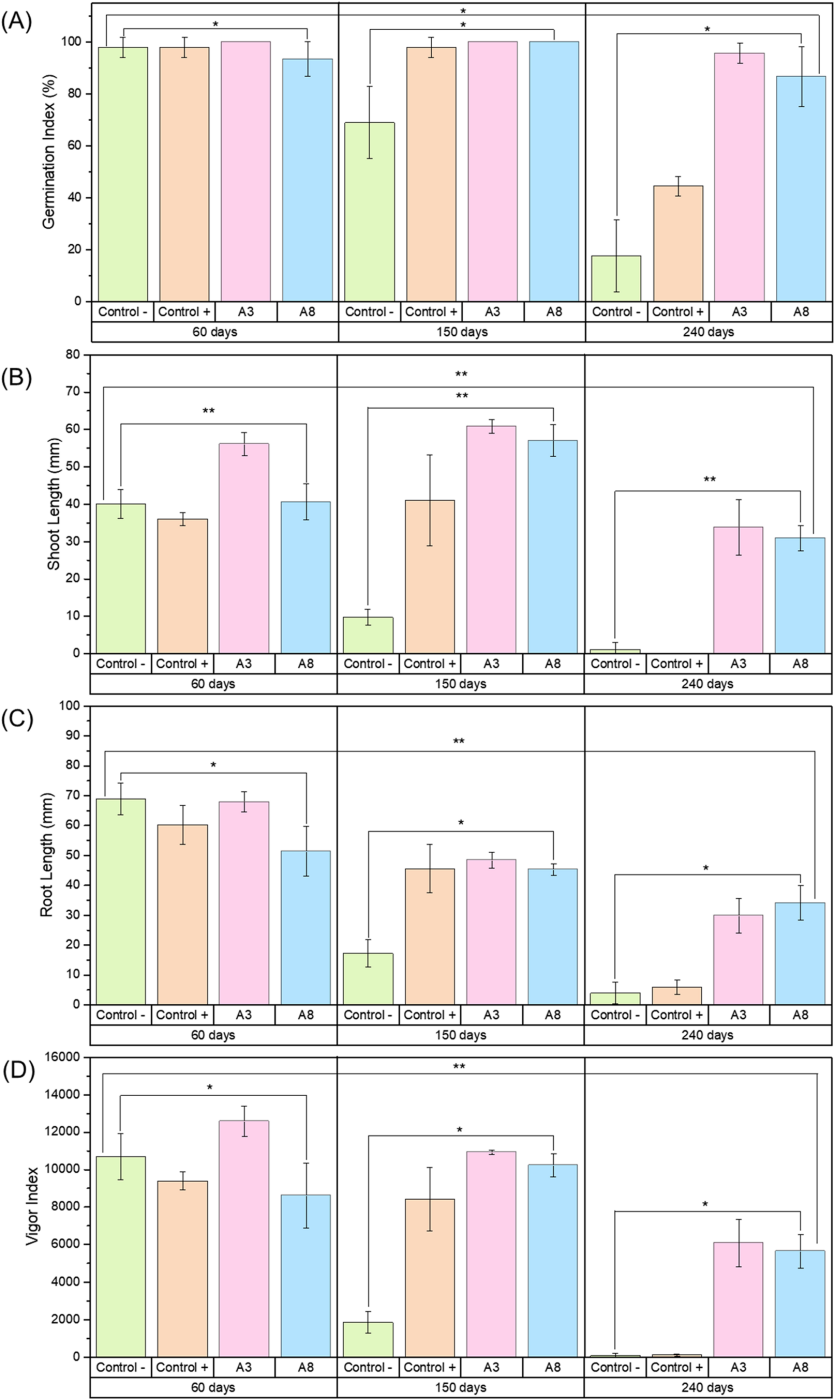
Parameters determined by germination assay: (A) Germination index; (B) shoot Length; (C) root Length; and (D) vigor Index, at different periods 60, 150, and 240 days after seed coating. Statistical differences between the groups or the storage time are represented with * (*p*-value >0.05) and ** (0.01 < *p*-value <0.05) by two-way ANOVA.

**Table 2:** Germination Assay Results with 60, 150, and 240 Days after Seed Coating^c^ ^,^ ^d^

Time After Seed Coating	Group	Average Dry Weight Before Germination (mg)	Average Fresh Weight (mg)	Average Dry Weight After Germination (mg)	Moisture Content (%)	Germination Index * (%)	Leaf Emergence (%)	Shoot Length^a^,^b^ (mm)	Root Length^b^ (mm)	Vigor Index^b^
60 days	Negative Control	2.18 ± 0.39	11.65 ± 2.57	1.59 ± 0.86	86.35 ± 2.71	97.78 ± 3.85	68.89 ± 3.85	40.07 ± 3.87	68.94 ± 5.39	10678.80 ± 1237.06
	Positive Control	2.14 ± 0.30	19.67 ± 1.89	1.61 ± 0.02	91.81 ± 1.89	97.78 ± 3.85	86.67 ± 0.00	36.00 ± 1.80	60.30 ± 6.48	9398.12 ± 475.64
	A3	2.34 ± 0.11	23.74 ± 1.41	1.70 ± 0.07	92.84 ± 1.41	100.00 ± 0.00	93.33 ± 0.00	56.13 ± 3.03	67.94 ± 3.37	12590.33 ± 803.41
	A8	2.38 ± 0.37	21.61 ± 0.68	1.67 ± 0.16	92.27 ± 0.70	93.33 ± 6.67	84.44 ± 3.85	40.60 ± 4.82	51.34 ± 8.32	8618.14 ± 1735.59
150 days	Negative Control	1.84 ± 0.20	4.77 ± 1.21	1.80 ± 0.18	62.26 ± 1.22	68.89 ± 13.88	15.56 ± 3.85	9.75 ± 2.16	17.28 ± 4.49	1865.59 ± 579.11
	Positive Control	1.85 ± 0.09	16.45 ± 4.13	1.71 ± 0.08	89.60 ± 4.13	97.78 ± 3.85	64.44 ± 13.88	41.05 ± 12.09	45.60 ± 8.16	8427.39 ± 1685.09
	A3	2.08 ± 0.21	25.09 ± 1.91	1.93 ± 0.21	92.31 ± 1.92	100.00 ± 0.00	86.67 ± 6.67	60.86 ± 1.85	48.42 ± 2.78	10928.00 ± 118.50
	A8	2.02 ± 0.10	24.32 ± 0.95	1.87 ± 0.09	92.31 ± 0.95	100.00 ± 0.00	86.67 ± 6.67	57.03 ± 4.21	45.29 ± 1.82	10231.00 ± 601.20
240 days	Negative Control	1.97 ± 0.25	2.63 ± 0.29	1.74 ± 0.03	33.84 ± 0.29	17.78 ± 13.88	2.22 ± 3.85	1.12 ± 1.95	3.98 ± 3.55	84.26 ± 132.56
	Positive Control	1.91 ± 0.12	2.83 ± 0.18	1.72 ± 0.02	39.22 ± 0.18	44.45 ± 3.85	0.00 ± 0.00	0.00 ± 0.00	5.96 ± 2.32	119.49 ± 48.97
	A3	2.06 ± 0.11	15.57 ± 2.52	1.71 ± 0.07	89.02 ± 2.52	95.55 ± 3.85	75.56 ± 3.85	33.85 ± 7.42	29.90 ± 5.82	6089.52 ± 1247.07
	A8	2.18 ± 0.05	13.46 ± 3.21	1.80 ± 0.09	86.63 ± 3.21	86.66 ± 11.55	60.00 ± 13.33	30.94 ± 3.32	34.12 ± 5.82	5648.27 ± 897.95

a Significant difference between groups (0.01 < *p*-value <0.05).

b Significant difference between the time after seed coating (0.01 < *p*-value <0.05).

c The results were subjected to a two-way ANOVA, considering groups and time as factors (Table S2—Supporting Information).

d * No significant difference (*p*-value >0.05).

As shown in Table 2, the moisture content significantly decreases over time in both control groups, indicating progressive water loss from the seeds. This moisture content fluctuation can affect germination performance by reducing metabolic and respiratory activities, compromising membrane integrity, increasing oxidative stress due to the accumulation of reactive oxygen species, and ultimately decreasing seed viability and germination rates,^14,26^ as will be demonstrated in the results below. After 200 days, the uncoated seeds probably experienced deterioration due to moisture loss and oxidative stress. In contrast, starch coatings reduce oxygen permeability and microbial attack, preserving seed viability for a longer period.

It is possible to observe, in Figure 7, that, after 60 days of storage, all groups presented similar germination indices within the margin of statistical error. Although germination percentages at 60 days were comparable, seedlings from coated seeds exhibited greater shoot length. Two mechanisms can explain this difference: first, the presence of urea within the coating likely provided an available nitrogen source during early seedling growth, enhancing shoot elongation without necessarily affecting the initial germination ratio;^27^ and second, the starch-based film is hydrophilic and increases moisture retention around the seed (supported by previous results), leading to a more favorable environment, as well as the glycerol, as a plasticizer, improves film integrity and reduces fissuring, limiting uncontrolled desiccation or pathogen ingress that could otherwise impair seedling growth.^28,29^ However, after 150 days, the negative control group exhibited a drastic decline in germination (dropping below 70%), while the other groups maintained stable values. At 240 days, this decline continued for the negative control group, reaching less than 20%, whereas the positive control group remained at 44%. These results demonstrate that the starch-based coatings not only do not interfere negatively with seed germination but also effectively provide protection. By acting as a physical barrier, the coatings extend the storage time of the seeds while maintaining germination rates comparable to those observed at earlier storage intervals. Practically, the starch–urea–glycerol film likely acted as a semipermeable barrier, reducing water and oxygen diffusion and thus limiting microbial ingress and seed respiration/oxidation, as previously reported.^30,31^

Even though the precise physicochemical mechanism governing water/oxygen diffusion reduction and the kinetics of urea release remain incompletely characterized in the literature, it is expected that the starch-based coating operates through a sequence of processes that evolve from storage to germination, as schematically illustrated in Figure 8: (I) During the storage period, the film forms a continuous biopolymer network around the seed, acting as a semipermeable barrier. The interconnected starch chains, plasticized with glycerol and urea, generate a tortuous diffusion pathway that slows the ingress of water vapor and oxygen, thereby reducing respiration and moisture-induced deterioration and preserving seed viability over time; (II) Upon exposure to water, the hydrophilic matrix absorbs moisture, leading to film hydration and swelling. This process increases chain mobility and weakens intermolecular interactions, particularly in more hydrophilic formulations; (III) As germination progresses, partial dissolution of the hydrated film enables the release of soluble components such as urea into the seed microenvironment. While the more stable A3 formulation maintains barrier integrity for longer periods, the more hydrophilic A8 film dissolves faster, facilitating earlier release and supporting initial seedling development. Although the individual contributions of diffusion, swelling, and dissolution were not quantitatively resolved, the observed behavior is consistent with a combined transport mechanism commonly reported for starch-based biopolymer films.^32^

**Figure 8:**
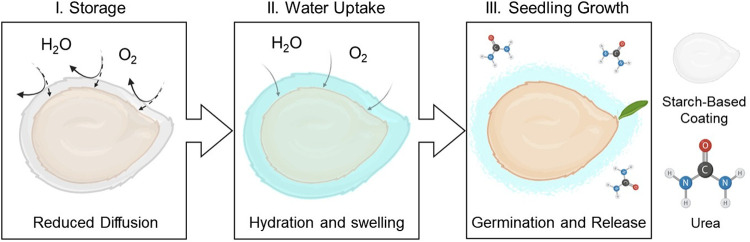
Schematic representation of the proposed mechanism of action of the starch-based seed coating: (I) formation of a semipermeable polymer barrier that slows water and oxygen diffusion during storage; (II) film hydration and swelling upon water uptake; and (III) partial dissolution of the matrix and release of soluble components, such as urea, during germination.

Regarding shoot and root lengths, all groups followed a similar trend over time. However, seeds coated with the A3 formulation consistently showed significantly higher growth, followed by those coated with A8. The control groups (positive and negative) presented significantly lower values. Notably, with increased storage time, the A8-coated seeds also maintained effective performance, likely due to their higher hydrophilicity and faster film dissolution, which supports early stage development.

The vigor index followed a pattern similar to the germination and growth parameters, as it is calculated based on both the germination index and seedling length. Over time, the coated seed groups (A3 and A8) maintained significantly higher vigor indices than both control groups, which approached values close to zero.

Finally, it is important to highlight that coating tomato seeds with either of the tested formulations, A3 or A8, represents a highly promising and cost-effective strategy. The use of abundant, biodegradable, and low-cost materials contributes to environmental sustainability while significantly improving seed performance. This approach has positive implications across multiple fields, including chemistry, biology, and especially agriculture.

Currently, commercially employed seed coating materials are predominantly based on synthetic film-forming polymers, such as poly­(vinyl alcohol) (PVA), acrylic and styrene-acrylate copolymers, and ethylene-derived polymers. Despite their good adhesion and mechanical stability, these systems typically rely on complex synthetic routes, involve higher production costs, exhibit limited or no biodegradability, and may contribute to the accumulation of microplastics in agricultural soils, raising increasing environmental and regulatory concerns. In this context, a coating formulation starch-based emerges as a highly attractive alternative, combining facile aqueous processing, low-cost and renewable raw materials, high biodegradability, low toxicity, and excellent soil compatibility. Altogether, these attributes position this system as a sustainable and efficient platform for next-generation seed coating technologies.

In summary, this study presents a proof of concept for the use of corn starch-based films plasticized with glycerol and urea as seed coating materials. Among the tested compositions, two formulations (A3 and A8) were selected, showing enhanced seed performance during storage under controlled conditions. Both coatings effectively preserved germination and vigor indices over extended periods (150 and 240 days), compared to the significant decline observed in control groups. Importantly, the A3 formulation exhibited higher thermal stability, a more homogeneous structure, and superior germination results, while the more hydrophilic A8 formulation facilitated early seedling development due to its faster dissolution. Although evaluated at laboratory scale, these findings demonstrate the feasibility of this approach and establish a foundation for future scale-up and field-level investigations. Overall, corn starch-based films can extend the storage time of tomato seeds while maintaining physiological integrity, and owing to their abundance, biodegradability, nontoxicity, and low cost, they represent a sustainable and practical strategy with strong potential for agricultural applications.

## Supplementary Material



## Notes

Visual aspects of corn starch-based films with different formulations; TGA curves and values of the thermal events for tomato seeds separated into four groups (negative and positive control, coated with A3 and A8 formulations); and final seedling development after 7 days of germination assay (PDF)
