# Supplementary material for: Starch-Based Film Coatings as a Strategy to Preserve Seed Viability during Storage
Source: ACS Omega. 2026 Jun 20;11(26):38652–62. doi: 10.1021/acsomega.6c01438 (PMC13347339; doi:10.1021/acsomega.6c01438)
Supplement: Supplementary file 1 [file ao6c01438_si_001.pdf]

Supporting Information

Starch-Based Film Coatings as a Strategy to Preserve Seed Viability During Storage

Giovana A. Parolin[1,2], Matheus C. R. Miranda[1], Tereza S. Martins[1], Marystela Ferreira[2], Laura O. Péres[1]

[1]Laboratory of Hybrid Materials – Chemistry Department – Federal University of São Paulo, Diadema – SP, Brazil, 09913-030

[2]Science and Technology Center for Sustainability – Federal University of São Carlos, Sorocaba – SP, Brazil, 18052-780

*Corresponding author: Laura O. Péres (laura.peres@unifesp.br)

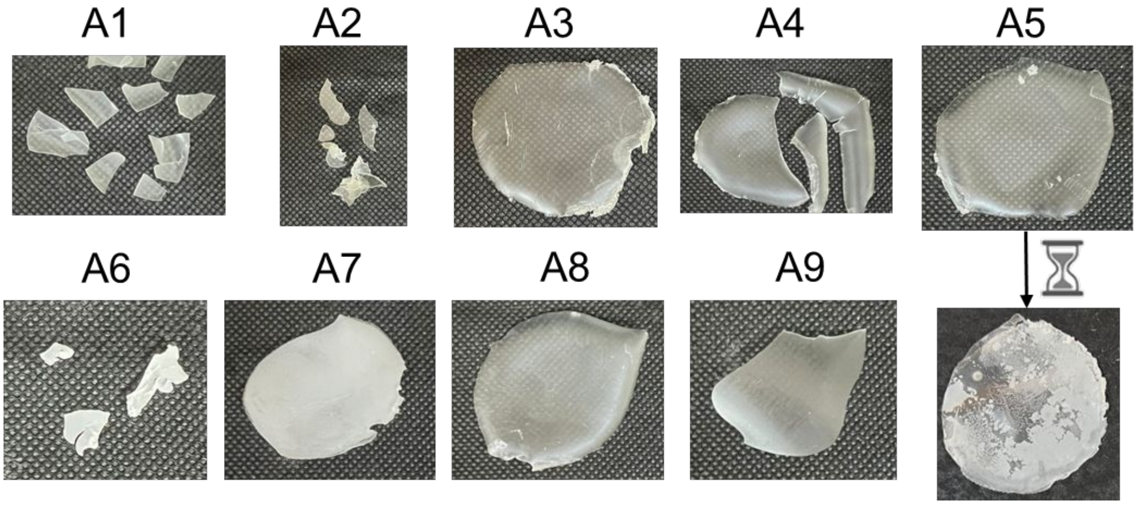

Figure S1. Visual aspect of corn starch-based films with different formulations, highlighting the porous structure with time by film A5.

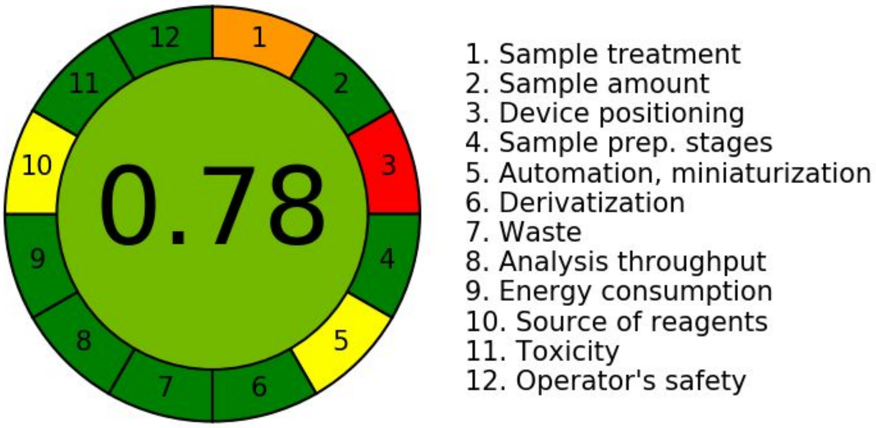

Figure S2. AGREE assessment of the proposed seed coating method, resulting in a high overall greenness score of 0.78, indicating good compliance with the principles of green chemistry.

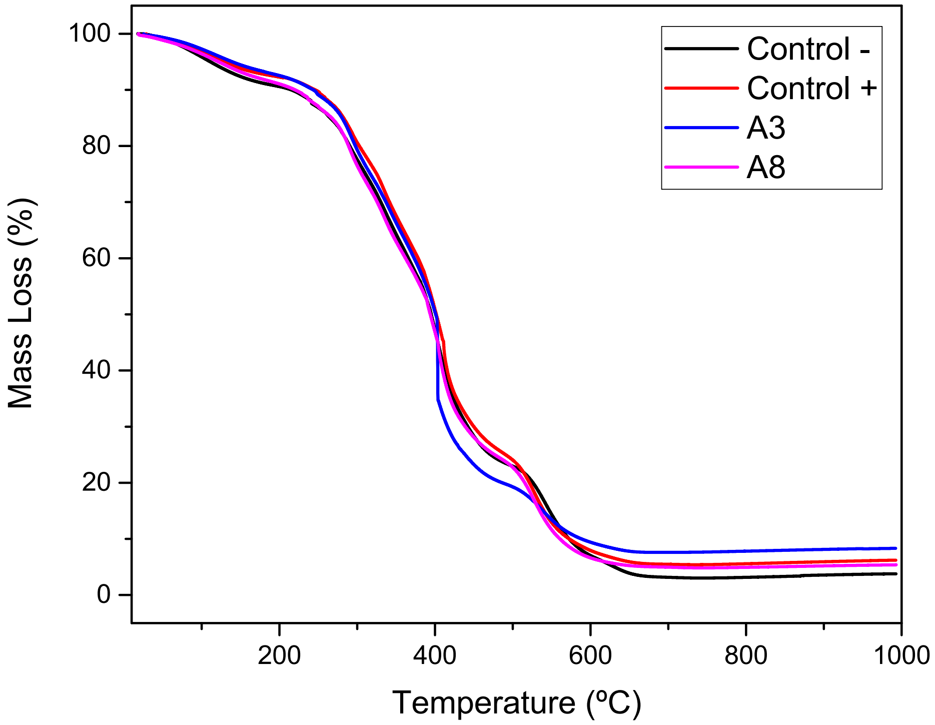

Figure S3. TGA curves for tomato seeds separated into four groups: Negative control (untreated seeds), positive control (immersed in water), and coated with starch formulations (A3 and A8). Conditions: From 30 ºC to 1000 ºC at a rate of 10 ºC/min, under a nitrogen atmosphere with a flow rate of 20 mL/min.

Table S1. Values of the thermal events observed in TGA curves for tomato seeds separated into four groups: Negative control (untreated seeds), positive control (immersed in water), and coated with starch formulations (A3 and A8).

**Sample**	**1º Event**			**2º Event**			**3º Event**			**Final Residue (%)**
	Ti (ºC)	Tf (ºC)	Mass Loss (%)	Ti (ºC)	Tf (ºC)	Mass Loss (%)	Ti (ºC)	Tf (ºC)	Mass Loss (%)	
Control -	30	185	9	185	510	69	510	680	19	3
Control +	30	185	7	185	500	69	500	680	19	5
A3	30	185	7	185	490	73	490	650	12	8
A8	30	185	8	185	488	68	488	660	19	5

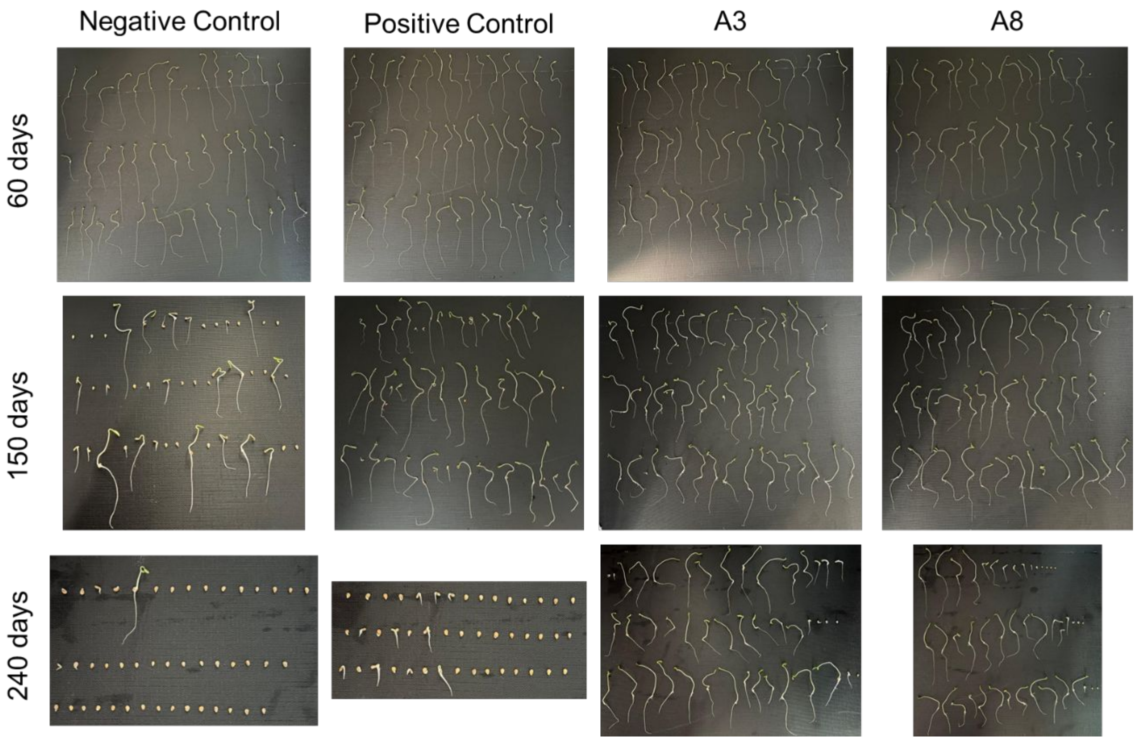

Figure S4. Final seedling development after 7 days of germination assay in untreated seeds (negative control), water-soaked seeds (positive control), and coated seeds (A3 and A8 formulations) after different storage periods (60, 150, and 240 days post-coating).

Table S2. Statistical significance (p-values) obtained by two-way ANOVA for seed performance parameters among control and coated groups (A3 and A8) at different storage times (60, 150, and 240 days).

Parameter	p-value A (Group)	p-value B (Time)
Germination Index	0.19	0.08
Shoot Length	**0.04**	**0.03**
Root Length	0.39	**0.01**
Vigor Index	0.13	**0.02**
